# Evaluation of reactive oxygen metabolites in patients with non-small cell lung cancer after chemotherapy

**DOI:** 10.1186/2049-6958-9-44

**Published:** 2014-08-29

**Authors:** Toru Wakabayashi, Tatsuo Kawashima, Yasuo Matsuzawa

**Affiliations:** 1 Department of Internal Medicine, Toho University Sakura Medical Center, 564-1 Shimoshizu, Sakura-shi, Chiba 285, Japan

**Keywords:** Chemotherapy, Lung cancer, Oxidative stress, Reactive oxygen metabolites, Reactive oxygen species

## Abstract

**Background:**

The aim of this study was to evaluate the level of reactive oxygen metabolites (ROMs) after chemotherapy in patients with non-small cell lung cancer (NSCLC) and its association with response to treatment.

**Methods:**

Fifty-eight untreated NSCLC patients and twenty-three healthy subjects were selected for the study. Patients received two courses of platinum-based chemotherapy and were evaluated for oxidative stress and treatment response. As a marker of reactive oxygen species, ROMs levels were measured using the d-ROMs test.

**Results:**

ROMs level (mean ± standard deviation) before chemotherapy in NSCLC patients (416 ± 135 U.CARR) was significantly elevated (p = 0.016) compared to normal healthy subjects (320 ± 59 U.CARR). Patients who responded to chemotherapy showed significantly decreased (p = 0.014) ROMs levels after chemotherapy, whereas patients who had stable disease or progressive disease showed no change in ROMs level (p = 0.387).

**Conclusions:**

NSCLC patients had significantly elevated ROMs levels before chemotherapy compared with normal healthy subjects. Chemotherapy may suppress ROMs production in responders but not in non-responders. ROMs level may be a predictor of clinical outcome in patients receiving chemotherapy for NSCLC.

## Background

Several mechanisms leading to increased oxidative stress in cancer patients have been proposed. Oxidative stress results in accumulation of reactive oxygen species (ROS) and reactive nitrogen species (RNS) such as hydroxyl radicals (OH^-^), superoxide anion radicals (O_2_^-^), lipid peroxyl radicals (LOO^-^), and nitrite radicals (NO^-^) [1-3]. Many anticancer drugs are also known to cause peroxidative damage to lipids and other biomolecules due to generation of free radicals [4-7]. The high level of oxidative stress generated during chemotherapy may overcome the antioxidant defenses of cancer cells, and affect anti-neoplastic activity [8]. Thus the antioxidant status of cancer patients may play an important role in their response to chemotherapy.

Direct measurement of ROS and free radicals in a standard laboratory is difficult owing to their biochemical instability. Recently, a method of measuring reactive oxygen metabolites (ROMs) in blood has been developed. This method is the d-ROMs (derivatives of reactive oxygen metabolites) test, which uses the Free Radical Analytical System (FRAS, Diacron, Grosseto, Italy) [9,10]. The main component of ROMs is hydroperoxide. Despite the moderate oxidative power, serum levels of hydroperoxides can be detected because of its relative stability compared with other parent free radicals.

The aim of this study is to evaluate the relationship between serum ROMs level and clinical response to chemotherapy in patients with non-small cell lung cancer (NSCLC).

## Methods

### Patients and controls

Fifty-eight NSCLC patients (aged 45–86 years; 43 men, 15 women), admitted to Toho University Medical Center Sakura Hospital between April 2007 and June 2010, were consecutively recruited for this study. All patients had histologically or cytologically confirmed NSCLC of clinical stage IIIB or IV at the time of diagnosis, and judged to be inoperable. Staging was based on the 6^th^ tumor node metastasis (TNM) staging system [11]. Patients with Eastern Cooperative Oncology Group (ECOG) performance status (PS) between 2 and 4 were excluded from the study. We also investigated 23 age-, sex- and smoking habit-matched healthy control volunteers (aged 47–81 years; 15 men, 8 women) who visited our hospital for health checkup between April 2007 and June 2010. None of the patients and healthy control volunteers had received antioxidant agents such as high-dose vitamin supplementation.

This study was approved by the Institutional Review Board of our institution, and written informed consent was obtained from all patients and control subjects.

### Measurement of reactive oxygen metabolites

Oxidative status was evaluated by measuring hydroperoxides in the serum using d-ROMs test. The test was performed using the Free Radical Analytical System 4 (FRAS 4; Wismerll Co. Ltd. Tokyo, Japan). The test is based on the concept that the amount of organic hydroperoxides present in serum is related to the free radicals from which they are formed. When the serum sample is dissolved in an acidic buffer, the hydroperoxides react with the transition metal ions liberated from the proteins in the acidic medium and are converted to alkoxy and peroxy radicals. These newly formed radicals oxidize the chromogen (*N*, *N*-diethyl-*para*-phenylendiamine) to produce a colored derivative. The concentration of this stable species can be determined by spectrophotometric procedures (absorption at 505 nm). The normal range is between 250 and 300 U.CARR. (Carratelli Units), where 1 U.CARR. corresponds to 0.8 mg/L H_2_O_2_. Values higher than 300 U. CARR suggest increased oxidative stress.

In all subjects, blood was collected from a peripheral vein. Twenty μl of blood sample and 1 mL of buffered solution (R2 reagent of the kit, pH 4.8) were gently mixed in a cuvette, and then 10 μl of chromogenic substrate (R1 reagent of the kit) was added into the cuvette. After mixing, the cuvette was centrifuged for 60 seconds at 37°C and incubated immediately in the thermostatic block of the analyzer for 5 min at 37°C. Then, absorbance at 505 nm was measured [9,10].

### Chemotherapy and evaluation of clinical response to treatment

The patients received one of the following three chemotherapy regimens: carboplatin/paclitaxel, carboplatin/gemcitabine, and cisplatin/gemcitabine. The carboplatin/paclitaxel regimen consisted of carboplatin at an area under the curve (AUC) of 5 mg/mL • min on day 1 and paclitaxel at 180 mg/m^2^ on day 1 every 4 weeks. The carboplatin and gemcitabine combination consisted of carboplatin AUC 5 mg/mL • min on day 1 plus gemcitabine 1000 mg/m^2^ on days 1 and 8 every 4 weeks. Carboplatin was replaced by cisplatin (80 mg/m^2^ on day 1) in the cisplatin/gemcitabine regimen. All patients received at least two courses of the respective regimens. Tumor response was evaluated according to the Response Evaluation Criteria in Solid Tumors (RECIST) [12], before chemotherapy and after two courses (8 weeks). ROMs levels in all patients were assessed before treatment (day 0). In 18 of 58 NSCLC patients, both tumor response and ROMs level were evaluated after receiving the second course of chemotherapy.

### Statistical analysis

The results are expressed as mean ± standard deviation (SD) unless stated otherwise. Levels of oxidative stress between two different groups were compared using *t -*tests, as the data were normally distributed. P less than 0.05 was considered to be statistically significant. All the above-mentioned analyses were performed using the SPSS II 11.0 for Windows software package (SPSS Japan Inc., Tokyo, Japan).

## Results

### Baseline patient characteristics and control subjects

Patient characteristics are summarized in Tables 1 and 2. The patient group consisted of 43 (74.1%) men and 15 women (25.9%), with a median age of 69.3 years (range, 45–86 years). Fifty patients (86.2%) had adenocarcinoma and 8 patients (13.8%) had squamous cell carcinoma. All patients had ECOG PS of 0 or 1, and had undergone no prior therapy. Forty-five patients (77.6%) were current or former smokers and 13 (22.4%) patients had never smoked (non-smokers). The ROMs levels measured by the d-ROMs test were significantly increased in squamous cell carcinoma patients (p = 0.032) and current/former smokers (p = 0.040) compared with adenocarcinoma patients and non-smokers, respectively. There were no significant differences in ROMs level between males and females, performance status 0 and 1, and clinical stages IIIB and IV.

**Table 1:** Characteristics of 58 patients with non-small cell lung cancer enrolled in the study

**Characteristic**	**Number**	**ROMs level (U.CARR)**	**p**
Gender			
Male	43	437 ± 144	
Female	15	356 ± 81	0.134
Histology			
Adenocarcinoma	50	395 ± 107	
Squamous cell carcinoma	8	549 ± 209	0.032*
Performance status			
0	41	419 ± 149	
1	17	410 ± 96	0.139
Clinical stage			
IIIB	21	405 ± 110	
IV	37	423 ± 148	0.407
Smoking history			
Current/former smoker	45	439 ± 143	
Non-smoker	13	340 ± 55	0.040*

ROMs levels are expressed as mean ± SD. Mean values were compared by unpaired*-t* test. *: p < 0.05. ROMs, reactive oxygen metabolites; U.CARR, Carratelli Units.

**Table 2:** Demographic data and ROMs levels (U.CARR) of non-small cell lung cancer patients before treatment and matched controls

**Characteristic**	**Non-small cell cancer patients(N = 58)**	**Controls (N = 23)**
	**(Histology: Adeno/ Squ =50/ 8)**	
Gender (male/female)	43/15	15/8
Age (years)	69.3 ± 10.4	65.6 ± 9.5
ROMs level (U.CARR)	416 ± 135	320 ± 59.0*

Age and ROMs level are expressed as mean ± SD. Mean values were compared by unpaired*-t* test.

*: p < 0.05 *vs* NSCLC patients*.* ROMs, reactive oxygen metabolites; U.CARR, Carratelli Units; Adeno, adenocarcinoma; Squ, squamous cell carcinoma.

**Figure 1:**
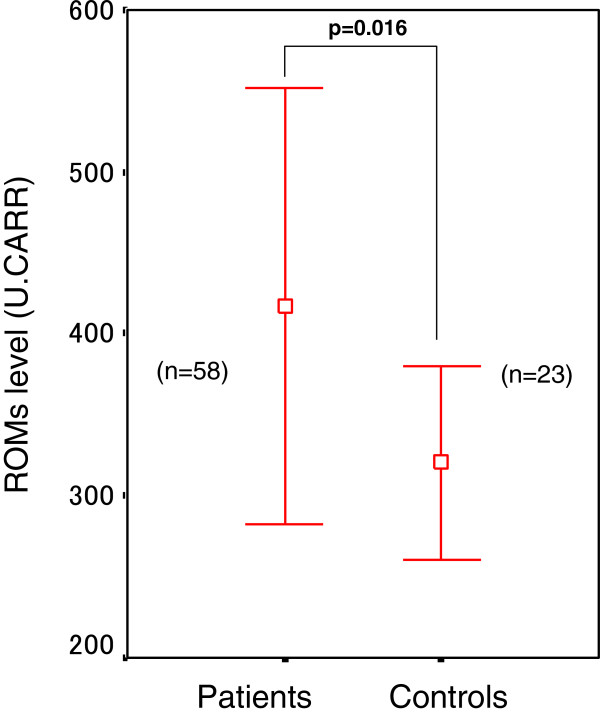
**ROMs levels (U. CARR) in control subjects and in patients with non-small cell lung cancer before treatment.** Data are expressed as mean ± SD. ROMs level was elevated significantly in patients before treatment compared with the control subjects (p *=* 0.016, unpaired *t*-test).

The control subjects consisted of 15 men (65.2%) and 8 women (34.8%), with a median age of 65.6 years (range, 47–81 years). Eighteen control subjects (78.2%) were current or former smokers and 5 control subjects (21.8%) had never smoked. There were no significant differences in age, gender ratio, and smoking habit between NSCLC patients and healthy controls. The ROMs level (mean ± SD) in NSCLC patients before treatment (416 ± 135 U. CARR) was elevated significantly (p = 0.016) compared with healthy controls (320 ± 59 U. CARR) (Table 2 and Figure 1).

### ROMs levels and response to chemotherapy

**Figure 2:**
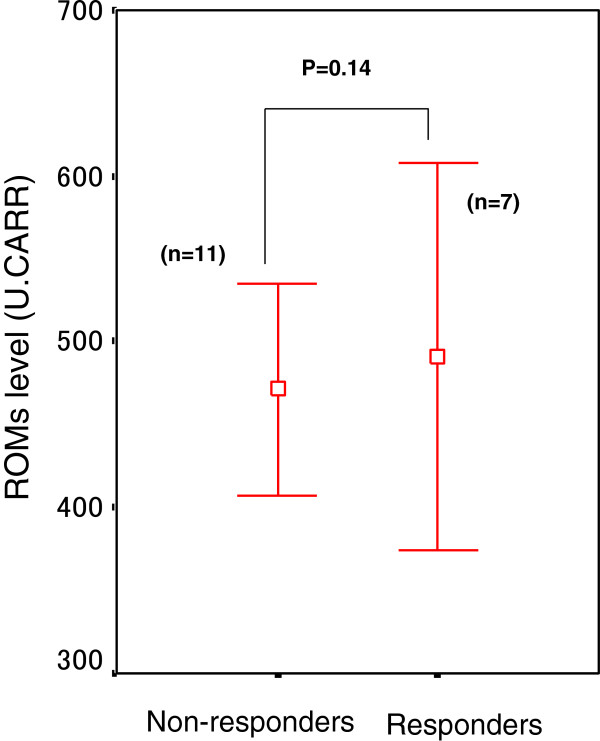
**Pretreatment ROMs levels (U. CARR) in patients with non-small cell lung cancer classified as responders (complete response/partial response) and non-responders (stable disease/progressive disease).** Data are expressed as means ± SD. No significant difference in ROMs level was observed between responders and non-responders (p *=* 0.14, unpaired *t*-test).

**Figure 3:**
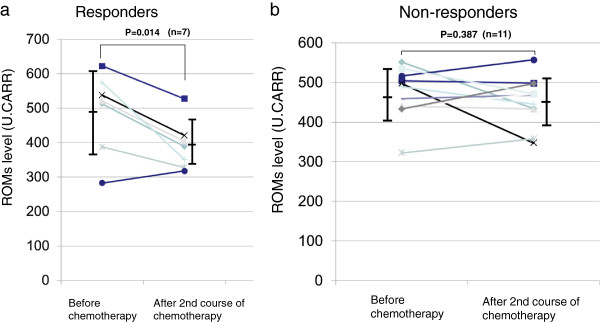
**ROMs levels (U. CARR) before and after 2**^**nd **^**course of chemotherapy in patients with non-small cell lung cancer classified as responders (complete response/partial response) and non-responders (stable disease/progressive disease).** Individual paired data and mean ± SD are shown. **a)** A significant decrease in ROMs level is observed in responders (p *=* 0.014, paired *t*-test). **b)** No significant difference in ROMs levels is found in nonresponders (p *=* 0.387, paired *t*-test).

In 18 patients evaluated after receiving the second course of chemotherapy, 7 patients (adenocarcinoma/squamous cell carcinoma; 5/2) were classified as responders (complete or partial response: CR/PR), while 11 patients (adenocarcinoma/squamous cell carcinoma; 9/2) were classified as non-responders (stable disease or progressive disease: SD/PD). Before chemotherapy, ROMs levels did not differ significantly between responders and non-responders (491 ± 116 vs. 471 ± 63 U. CARR; p = 0.14) (Figure 2). After the second course of chemotherapy, a significant decrease in ROMs level was observed in responders (CR/PR) (491 ± 116 vs. 391 ± 71 U. CARR, p = 0.014) (Figure 3a), while no change in ROMs level was found in non-responders (SD/PD) (471 ± 63 vs. 452 ± 60 U. CARR; p = 0.387) (Figure 3b).

### Follow up after the second course of chemotherapy

Eleven non-responders discontinued the chemotherapy regimens after the second course. Among seven responders (PR/CR), two patients discontinued chemotherapy after the second course; one achieved down-staging and underwent surgical operation, and the other developed drug-induced interstitial pneumonia and died. One patient underwent three courses of chemotherapy. This patient deteriorated to PD after the third course, and ROMs level increased from 328 to 358 U.CARR. Treatment was changed to oral gefitinib because of abnormal epidermal growth factor receptor level. After chemotherapy was restarted, her condition improved to PR, and ROMs level also decreased from 358 to 268 U.CARR. The remaining four patients were able to complete four or more courses of chemotherapy and maintained PR/CR. In one of these four patients, ROMs level was evaluated after the fourth course. Although ROMs level increased from 283 to 318 U.CARR after the second course of chemotherapy, the level decreased to 241U.CARR after the fourth course.

### Other tumor markers

In addition to ROMs levels, we measured carcinoembryonic antigen (CEA) levels in patients with adenocarcinoma and squamous cell carcinoma antigen (SCC) levels in patients with squamous cell carcinoma, as markers for monitoring chemotherapy for NSCLC. Among seven responders, five had adenocarcinoma and two had squamous cell carcinoma. Three out of five patients with adenocarcinoma had elevated CEA levels before chemotherapy, and the level decreased in one and increased in two patients after the second course of chemotherapy. The other two patients with normal CEA levels before chemotherapy showed changes within the normal range even after the second course of chemotherapy. SCC levels were elevated before chemotherapy in both patientswith squamous cell carcinoma, and the level decreased in one patient and increased in the other after the second course of chemotherapy.

## Discussion

The main findings in our study are that: (1) ROMs level was significantly elevated in NSCLC patients before chemotherapy compared with age-, sex- and smoking habit-matched healthy controls; and (2) ROMs level decreased significantly in responders but was unchanged in non-responders after the second course of chemotherapy.

Recently, the concept of ‘persistent oxidative stress in cancer’ has been hypothesized to partly explain the characteristics of tumor biology such as activated transcription factors and proto-oncogenes, genomic instability, chemotherapy-resistance, as well as invasion and metastasis [13]. What kinds of cells produce ROS in cancer patients? Mounting evidence suggests that malignant cells produce a large amount of hydrogen peroxide [14]. Toyokuni et al*.*[13] reported that cancer cells themselves produce ROS in human renal cell carcinoma. This mechanism is supported by previous reports that a large amount of hydrogen peroxide is produced *in vitro* without exogenous stimulation in several human carcinoma cell lines, and that the antioxidant system is suppressed in cancer cells [15,16].Inokuma et al*.*[17] reported that serum ROS level was elevated in proportion to tumor invasion and correlated positively with tumor size, and they suggested that one possible therapeutic strategy would be to increase ROS scavenging against target ROS-triggered signal transduction. In our study, ROMs level was significantly elevated in NSCLC patients compared with healthy controls, and the level decreased in responsive NSCLC patients, but was not changed in non-responsive patients after the second course of chemotherapy. These changes in ROMs level may support the findings of the above studies.

In NSCLC patients, the ROMs levels were significantly higher in patients with squamous cell carcinoma and in current or former smokers compared with adenocarcinoma (p = 0.032) and non-smokers, respectively (p = 0.040). In the present study, all patients with squamous cell carcinoma were smokers, while 13 out of 50 patients with adenocarcinoma were non-smokers. ROMs levels have been reported to increase in smoking, diabetes and dialysis [18]. Our findings are consistent with this report.

Indeed, accumulating evidence suggests that evaluation of oxidative stress in NSCLC patients may predict the prognosis of the patients [19]. On the other hand, there is also a question on which markers best indicate the general condition of oxidative stress. In this study, we measured ROMs to evaluate the status of oxidative stress. Several methods may be used to evaluate oxidative stress, including (1) measurement of active oxygen species, (2) detection of oxidized DNA and lipids, and (3) quantification of antioxidants. However, the actual measurement and evaluation of these compounds involve various problems, because a large number of substances are involved in the oxidation-reduction system. Moreover, these substances interact in a complex manner through cross-talk. Therefore, interpretation of the results requires consideration of the type of sample, sampling site, substance measured, and method of measurement. Accurate measurement may be difficult because the targets of measurement are often unstable substances and the difference from background level is often very small. Instead of directly measuring ROS or free radicals, it is now possible to measure the hydroperoxide concentration in the blood produced by active oxygen or free radicals, using a colorimetric method. The measured concentration is considered to be directly proportional to the quantity of ROMs produced by active ROS and free radicals. Recent reports suggest that ROMs in serum is a reliable biomarker, and that the concentration of ROS in serum has a high positive correlation with serum ROMs levels measured by the d-ROMs test [20-23].

Oxidative stress causes injury to cells, induces gene mutation, and is involved in carcinogenesis by influencing intracellular signal transduction and transcription factors directly or indirectly via oxidants. Easy and accurate methods of measuring oxidative stress in the human body are indispensable for investigating the association with disease and for applying the results of research to clinical practice. Therefore, measuring serum ROMs levels may allow quantitative evaluation of the status of oxidative stress in NSCLC patients and the effect of chemotherapy.

This study had a relatively small sample size and evaluated the effect after a short course of chemotherapy. We have been very cautious in interpreting our results. Nevertheless, our results suggest that ROMs level may be a predictor of clinical outcome in patients receiving chemotherapy for NSCLC. Further research on a larger number of patients is needed to validate the present finding.

## Conclusions

This report is the first to demonstrate an association between ROMs level and response to chemotherapy in patients with NSCLC. We found that in NSCLC patients, ROMs level before chemotherapy was significantly elevated compared with normal healthy subjects, and chemotherapy might suppress ROMs production in responders but not in non-responders. ROMs may be a predictor of clinical outcome in NSCLC patients receiving chemotherapy.

## Abbreviations

CR: Complete response; NSCLC: Non-small cell lung cancer; PR: Partial response; PD: Progressive disease; ROM: Reactive oxygen metabolites; SD: Stable disease.

## Competing interests

The authors declare that they have no competing interests.

## Authors’ contributions

TW participated in the design of the study and interpretation of data, provided clinical care for patients, and drafted the manuscript. TK participated in ROMs assays, performed statistical analysis, and reviewed the manuscript critically. YM participated in clinical care of patients and interpretation of data, and reviewed the manuscript critically. All authors have read and approved the final manuscript.
